# Strengthening of Existing Episodic Memories Through Non-invasive Stimulation of Prefrontal Cortex in Older Adults with Subjective Memory Complaints

**DOI:** 10.3389/fnagi.2017.00401

**Published:** 2017-12-05

**Authors:** Rosa Manenti, Marco Sandrini, Elena Gobbi, Chiara Cobelli, Michela Brambilla, Giuliano Binetti, Maria Cotelli

**Affiliations:** ^1^Neuropsychology Unit, IRCCS Istituto Centro San Giovanni di Dio Fatebenefratelli, Brescia, Italy; ^2^Department of Neurorehabilitation Sciences, Casa di Cura Privata del Policlinico, Milan, Italy; ^3^Molecular Markers Laboratory, IRCCS Istituto Centro San Giovanni di Dio Fatebenefratelli, Brescia, Italy; ^4^MAC Memory Center, IRCCS Istituto Centro San Giovanni di Dio Fatebenefratelli, Brescia, Italy

**Keywords:** SMC, tDCS, reconsolidation, prefrontal cortex, episodic memory

## Abstract

Episodic memory is critical to daily life functioning. This type of declarative memory declines with age and is the earliest cognitive function to be compromised in Alzheimer’s disease (AD). Subjective memory complaints are commonly reported by older adults and have been considered a risk factor for developing AD. The possibilities for prevention of memory disorders in older adults have increased substantially in recent years. Previous studies have shown that anodal transcranial Direct Current Stimulation (tDCS) applied over the left lateral prefrontal cortex (PFC) after a contextual reminder strengthened existing verbal episodic memories, conceivably through reconsolidation, in elderly people. In this study, we hypothesized that anodal tDCS applied over the left lateral PFC after a contextual reminder would improve delayed memory retrieval relative to placebo (sham) stimulation in elderly individuals with SMC. Twenty-two subjects learned a list of words. Twenty-four hour later, tDCS (anodal or placebo) was applied over the left lateral PFC after a contextual reminder. Memory retrieval was tested 48h and 30 days later. These findings showed that anodal tDCS over the left lateral PFC strengthened existing episodic memories, a behavioral effect documented by improved recognition up to 30 days, relative to placebo stimulation. This study suggests that tDCS after a contextual reminder can induce long-lasting beneficial effects by facilitating the consolidation processes and opens up the possibility to design specific non-invasive interventions aimed at preventing memory decline in this at-risk population.

## Introduction

There is evidence that episodic memory declines with age (Spencer and Raz, 1995; Balota et al., 2000; Salthouse, 2010; Rhodes and Katz, 2017; Solesio-Jofre et al., 2017). Subjective memory complaints (SMC) refer to self-reports of memory decline with objective memory performance in the normal range (Vannini et al., 2017). Older adults generally report SMC with a prevalence estimated from 22 to 56% of this population (Jorm et al., 1994; Geerlings et al., 1999; Montejo et al., 2011). The concept of SMC is highly significant to the field of aging because this condition is a risk factor for developing Alzheimer’s disease (AD) (Glodzik-Sobanska et al., 2007; Jessen et al., 2010; Dubois et al., 2014; Jessen et al., 2014; Vannini et al., 2017). In addition, SMC is a criteria for the diagnosis of Mild Cognitive Impairment (MCI) due to AD (Albert et al., 2011).

Episodic memory is critical to daily life functioning (Tulving, 1983) and several clinical works have reported that this type of long-term memory relies on the integrity of the medial temporal lobe (MTL) (Dickerson and Eichenbaum, 2010). In addition, numerous studies reported that the prefrontal cortex (PFC) and MTL–PFC interactions are important for episodic memory processes (Simons and Spiers, 2003; Szczepanski and Knight, 2014; Eichenbaum, 2017). Evidence supporting the critical role of lateral PFC in episodic memory along the life span comes from clinical neuropsychology (Jetter et al., 1986; Janowsky et al., 1989a,b; Incisa Della Rocchetta and Milner, 1993; Eslinger and Grattan, 1994; Gershberg and Shimamura, 1995; Mangels, 1997; Alexander et al., 2003; Duarte et al., 2005), functional magnetic resonance imaging (Cabeza et al., 1997, 2000; Fletcher and Henson, 2001; Dennis et al., 2007, 2008), and transcranial magnetic stimulation studies (Rossi et al., 2001, 2004, 2011; Sandrini et al., 2003; Floel et al., 2004; Kohler et al., 2004; Innocenti et al., 2010; Manenti et al., 2010a, 2011, 2012; Gagnon et al., 2011; Blumenfeld et al., 2014).

Transcranial Direct Current Stimulation (tDCS, Dayan et al., 2013; Parkin et al., 2015) has been mainly used in cognitive neuroscience to modulate cognitive functions, an issue of potential clinical impact (Sandrini and Cohen, 2013, 2014; Tatti et al., 2016; Birba et al., 2017; Lefaucheur et al., 2017).

Anodal tDCS applied over the left lateral PFC during retrieval improved recognition performance (Manenti et al., 2013), applied after consolidation with a contextual reminder (Sandrini et al., 2014) or during learning (Sandrini et al., 2016) improved delayed recall in older adults. We also directly compared the two studies, in which the same paradigm was used (Sandrini et al., 2014, 2016), to determine which of the tDCS protocols would induce longer lasting effects. We found that anodal tDCS after consolidation with a contextual reminder induced longer-lasting effects (up to 30 days) on episodic memory, conceivably through reconsolidation, relative to anodal tDCS during learning (Manenti et al., 2016).

The consolidation model assumes that new memories are fragile (i.e., vulnerable to interference) for few hours after the encoding. With the passage of time, these memories stabilize and become resistant to interference (Mcgaugh, 2000). However, accumulating evidence has shown that consolidated memories can return to fragile states during retrieval or by a reminder cue and must consolidate again or reconsolidate (Dudai, 2012). Importantly, during this time-limited reconsolidation window, existing memories can be modified (e.g., strengthened) through behavioral means, pharmacological agents, or non-invasive brain stimulation techniques (Forcato et al., 2014; Sandrini et al., 2015).

In the present randomized, double-blind study, we tested the hypothesis that anodal tDCS applied over the left lateral PFC after a contextual reminder would improve delayed memory retrieval relative to placebo (sham) stimulation in elderly people with SMC.

On Day 1, older adults learned a list of 20 words. Twenty-four hours (h) later, tDCS (anodal or placebo) was applied after a contextual reminder. Memory retrieval (i.e., free recall and recognition) was tested 48 h and 30 days later. Based on previous findings showing improved memory performance up to 30 days (Sandrini et al., 2014), the primary endpoint measure was the change in memory performance tested 30 days after the learning session.

## Materials and Methods

### Participants

Twenty-two older individuals with SMC (14 females and 8 males; mean age = 74.5 ± 5.9 years; mean education = 9.9 ± 3.8 years) took part in the experiment. All of the subjects had normal or corrected-to-normal vision and were native Italian speakers. All participants were evaluated every 6 months for at least 12 months to obtain natural history data prior to be enrolled in the study.

The following were the inclusion and exclusion criteria:

– Inclusion: persons aged 60 or over, education between 5 and 18 years, Mini Mental State Examination (MMSE) score from 27 to 30 (Folstein et al., 1975), a score of more than 1.0 SD at Everyday Memory Questionnaire (EMQ) above the mean score obtained in a group of healthy older participants (mean 37.3, SD 8.4; Manenti et al., 2016), normal objective memory performance on neuropsychological tests, normal objective cognitive performance in all the administered tests, normal scores in functional assessment, absence of mood and anxiety disorders, absence of criteria for a diagnosis of dementia according to DSM-V (American Psychiatric Association, 2014).
– Exclusion: history of neurologic or major psychiatric disorder, history of head trauma with loss of consciousness, contraindications for tDCS (i.e., metal in the head and history of seizures), severe cardiovascular disease, use of medications that affect cognitive functions, alcohol or substance abuse. In addition, cerebrovascular disease or presence of cortical infarct, multiple lacunar strokes, or extensive white matter hyperintensities assessed using structural MRI.

Prior to being enrolled in the study all participants were informed about the study and the possible risks of tDCS and signed a written informed consent after a safety screening. The protocol was approved by the local Human Ethics Committee of IRCCS Fatebenefratelli of Brescia, Italy.

#### Assessment Procedures

##### SMC measures

The 28-item version of the EMQ was used for the evaluation of memory complaints (Sunderland, 1984;Calabria et al., 2011) (see **Table 1** for details).

**Table 1:** Demographic characteristics and clinical and neuropsychological assessment.

	AtDCS (*n* = 11)	PtDCS (*n* = 11)	Cut-off	*p*-value
Age (years)	75.9 (7.1)	73.1 (4.7)		ns
Gender (male/female)	4/7	4/7		
Education (years)	9.6 (3.6)	10.3 (4.3)		ns
EHI	98.5 (5.1)	87.5 (29.6)		ns
**Mood and Anxiety Assessment**				
Geriatric Depression Scale (GDS)	5.5 (4.3)	5.8 (2.9)	<11	ns
State-Trait Anxiety Inventory (STAI)				
STAI-State	43.8 (3.7)	44.4 (4.2)		
STAI-Trait	43.2 (6.6)	44.8 (4.4)		
**Functional Assessment**				
Activities of Daily Living (ADL)	0 (0)	0.1 (0.3)		ns
Instrumental Activities of Daily Living (IADL)	0.1 (0.3)	0.1 (0.3)		ns
**Cognitive Reserve Index (CRI)**				
CRI-Total Score	115.9 (11.9)	112.3 (16.9)		ns
CRI-Education	98.0 (20.9)	101.0 (21.3)		ns
CRI-Working Activity	97.4 (14.3)	101.7 (14.6)		ns
CRI-Leisure Time	140.3 (29.0)	124.9 (22.8)		ns
**Everyday Memory Questionnaire (EMQ)**	66.4 (25.8)	80.0 (26.0)		ns
**Screening for dementia**				
MMSE	28.0 (1.7)	27.5 (2.0)	≥24	ns
**Non-Verbal Reasoning**				
Raven’s colored progressive matrices	27.3 (3.3)	26.8 (3.8)	>17.5	ns
**Language**				
Token Test	32.0 (2.5)	32.0 (1.9)	>26.25	ns
Fluency, phonemic	31.1 (7.4)	32.5 (11.1)	>16	ns
Fluency, semantic	33.8 (11.9)	37.5 (7.5)	>24	ns
**Memory**				
Digit Span	5.5 (1.0)	5.3 (1.0)	>4.25	ns
Story Recall	9.8 (3.2)	11.6 (4.4)	>7.5	ns
AVLT (Immediate recall)	38.1 (11.7)	38.4 (9.3)	>28.52	ns
AVLT (Delayed recall)	7.6 (2.4)	7.6 (3.0)	>4.68	ns
Rey-Osterrieth complex figure, recall	12.5 (5.4)	12.8 (3.1)	>9.46	ns
**Praxis**				
Rey-Osterrieth complex figure, copy	30.3 (2.5)	29.7 (4.4)	>28.87	ns
**Executive functions**				
Trial Making Test-A (seconds)	52.8 (18.7)	41.0 (11.4)	<94	ns
Trial Making Test-B (seconds)	168.7 (79.4)	135.7 (49.2)	<283	ns

^∗^*Raw scores are reported (SD between blankets). AtDCS, Anodal tDCS; PtDCS, Placebo tDCS; EHI, Edinburgh Handedness Inventory; MMSE, Mini Mental State Examination; AVLT, Rey Auditory Verbal Learning Test; *p*-value: comparison between Anodal and Placebo groups, ns: not significant. Cut-off scores according to Italian normative data are reported*.

##### Neuropsychological assessment

The participants completed a MMSE (Folstein et al., 1975) and a neuropsychological evaluation in order to verify the absence of any objective cognitive deficit. All the tests were administered and scored according to standard procedures (Lezak et al., 2012) (see **Table 1** for details).

##### Functional assessment

Functional abilities were evaluated using activity of daily living (ADL) and instrumental activity of daily living (IADL) scales (Katz, 1983; Lawton and Brody, 1988).

##### Cognitive reserve questionnaire

Cognitive Reserve was investigated using the Cognitive Reserve Index questionnaire (CRIq) which offers a standardized measure of the cognitive reserve accumulated by individuals across their lifespan (Nucci et al., 2012).

##### Mood and anxiety measures

The 30-items version of the Geriatric Depression Scale (GDS; Yesavage et al., 1983) and the State-Trait Anxiety Inventory (STAI; Spielberger et al., 1983) were administered in order to exclude symptoms of depression and anxiety (Yates et al., 2015, 2017). The results of these assessments are presented in **Table 1**.

### Procedure

This protocol was almost identical to that used in our previous study with healthy older adults (Sandrini et al., 2014). There were four sessions on four different days: Day 1 (learning session), Day 2 (24 h later), Day 3 (48 h later) and Day 30 (30 days later). Participants knew that they would have to memorize a list of twenty words on Day 1 and that 24 h later they would receive a 15 minutes (min) session of tDCS. No information was given to them regarding the two retrieval sessions (i.e., Day 3 and Day 30). Twenty concrete words were selected from the “Corpus e Lessico di Frequenza dell’Italiano Scritto (CoLFIS)” (Laudanna et al., 1995). The words were balanced according to variables known to influence memory performance.

On Day 1, the experimenter pulled out one item at a time at random (i.e., a word written on piece of cardboard) from a white bag. Participants were asked to remember the words and then to place the cardboards in a blue bag. After all 20 words were placed into the bag, the experimenter asked the participants to recall the words. The procedure was repeated five times. Before the next learning trial, the words were mixed and placed in the white bag again. Participants filled in a memory strategies questionnaire (Manenti et al., 2010b) at the end of the experimental session.

Twenty-four hours later (Day 2), the same experimenter, in the same experimental room of Day 1, showed to the participants the empty blue bag and asked, “Do you remember this bag and what we did with it yesterday?”. Participants were asked to describe what they did on Day 1, but they were stopped if they started to recall the words learned. Participants received tDCS (anodal or sham) 10 min after the reminder because the reconsolidation process seems to begin about 10 min after memory reactivation (Monfils et al., 2009). It has been shown that existing memories are automatically reactivated if the participants return to the same experimental room of Day 1 (Hupbach et al., 2008; Sandrini et al., 2013).

**FIGURE 1:**
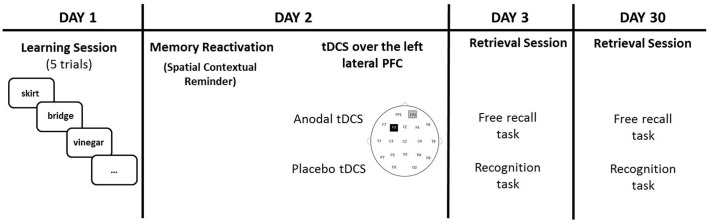
Participants learned 20 words on Day 1. On Day 2 (24 h later), tDCS (Anodal or Placebo) was applied over the left lateral PFC (anode over F3 and cathode over the right supraorbital area) after a spatial contextual reminder. Memory retrieval (free recall and recognition) was tested 48 h later (Day 3) and 30 days later (Day 30).

On Day 3, the experimenter asked the participants to recall the words learned during Day 1 (free recall task). When participants indicated that they could not remember any more words, the experimenter engaged the participants in an old/new recognition test that consisted in the written presentation of the 20 learned words along with 20 new words. Targets and new words were showed one at a time in a randomized order. Length, frequency and imageability of these words were balanced across lists. On Day 30, the procedure was the same of Day 3, but a nother set of new words was presented during the old/new recognition test (see **Figure 1** for a graphical representation).

#### tDCS Application

A tDCS stimulator (BrainStim, EMS, Bologna, Italy^1^) delivered constant current through two saline-soaked sponge electrodes (7 cm × 5 cm) at low intensity (1.5 mA). The current density (0.043 mA/cm^2^) was kept below the safety limits (Bikson et al., 2016; Antal et al., 2017). To reduce contact impedance, an electroconductive gel was applied under the two electrodes before the montage as done in previous studies (Manenti et al., 2013; Sandrini et al., 2014, 2016).

The study was double-blind. Active or placebo stimulation mode was selected by manual entering different codes, distributed by the principle investigator of the study, which activated either sham or active stimulation. The experimenter that applied tDCS could not notice any difference between active and sham tDCS.

**FIGURE 2:**
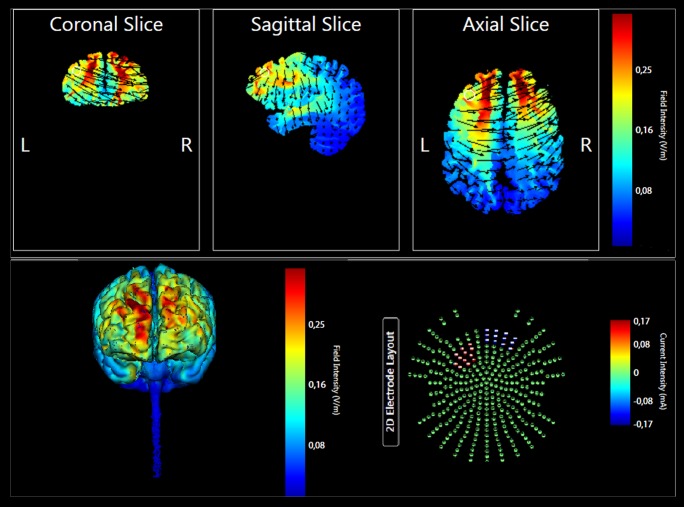
Current flow model of tDCS montage (anode over F3 and cathode over the right supraorbital area), using two 7 × 5 sponge pads represented in coronal, sagittal, and transverse views from the Male 1 model in the Soterix HD Targets software (Soterix Medical). Arrows represent direction of current flow.

The enrolled participants were randomly assigned to the Anodal tDCS (*n* = 11) or Placebo tDCS groups (*n* = 11). The targeted region was the left lateral PFC. The anode electrode pad was placed over F3, according to the 10–20 EEG international system for electrode placement, and the cathode electrode pad was placed over the right supraorbital area as done in previous studies (Manenti et al., 2013; Sandrini et al., 2014, 2016). See **Figure 2** for a graphical representation of the computerized modeling of tDCS-induced current flow in the brain according to these parameters (Soterix Medical^1^). In the anodal tDCS, the current was applied for 15 min (with a ramping period of 10 s) at the beginning and at the end of the tDCS session). In the placebo tDCS, the current was turned off 10 s after the beginning of the stimulation and was turned on for 10 s at the end of the stimulation period. With this procedure, it is difficult for participants to distinguish between active and sham stimulation (Manenti et al., 2013). Potential side effects and perceptual sensations induced by tDCS were assessed with a questionnaire after the stimulation session (Fertonani et al., 2015).

### Statistical Analyses

Demographic, clinical and neuropsychological variables, sensations induced by tDCS, cognitive reserve, SMC and memory strategy used were compared between the anodal and placebo groups using parametric (*t*-test) analyses.

The primary endpoint measure was memory performance at Day 30 (free recall and recognition). Accordingly, we analyzed our primary outcome using two-tailed independent *t*-tests comparing the two groups for the percentage of correctly recalled words on free recall at Day 30 and for the hit-false alarms rate on recognition at Day 30 (Bonferroni corrected for the number of comparisons, *p* = 0.05/2 = 0.025).

Moreover, we added a further analysis to explore changes on memory performance (free recall and recognition) at different time points (Day 3 and Day 30) in the two experimental groups (Anodal tDCS and Placebo tDCS). Thus, two mixed ANOVA models were adopted to analyze the dependent variables ‘percentage of correctly recalled words on free recall’ and ‘hit-false alarms rate on recognition’ at Day 3 and Day 30 including one within-subjects variable “Time” (Day 3 and Day 30) and one between-subjects variable “Group” (Anodal and Placebo).

Statistical analyses were performed using Statistica software^2^ (version 10). Statistical power and Effect Sizes (Cohen’s *d*) analyses were estimated using GPower 3.1 (Faul et al., 2007).

## Results

### Sample Characteristics

No differences were found between groups for demographic variables and for neuropsychological assessment (see **Table 1** for details). Moreover, no differences were observed between the Anodal and Placebo groups (see **Table 1**) for cognitive reserve (*t* = 0.58, *p* = 0.57), GDS (*t* = 0.23, *p* = 0.82), STAI – State (*t* = 0.33, *p* = 0.74), STAI – Trait (*t* = 0.69, *p* = 0.50), and EMQ (*t* = 1.23, *p* = 0.23). Importantly, none of the participants showed a pathological performance in an assessed cognitive ability and no subject reported mood and anxiety disorder. No differences were found between the Anodal and Placebo groups in the strategies questionnaire (Anodal tDCS group: 6.5, SD 3.7, Placebo tDCS group: 6.7, SD 3.1; *t* = 0.18, *p* = 0.86).

The tDCS sensations scores reported by the Anodal and Placebo groups were similar (Anodal tDCS group: 1.09, SD 0.7, Placebo tDCS group: 1.45, SD 0.8; *t*(20) = 1.12, *p* = 0.27).

### Experimental Memory Task

Participants correctly recalled on average 58.2% (SD 13.9) of the words after the last learning trial of Day 1 (Anodal = 60.4%, SD 12.3; Placebo = 56.0%, SD 16.3). There were no significant differences in the numbers of words correctly recalled between the Anodal and Placebo groups [*t*(20) = 0.74, *p* = 0.47].

Regarding the performance at Day 3, the mean percentage of words correctly recalled was 21.8% (SD 12.3) in the Anodal group and 14.1% (SD 9) in the Placebo group. In the recognition task, the hit-false alarms score was 16.2 (SD 3.6) in the Anodal group and 9.1 (SD 3.3) in the Placebo group.

At Day 30, the mean percentage of words correctly recalled was 10.9% (SD 10.8) in the Anodal group and 9.6% (SD 11.4) in the Placebo group. The hit-false alarms score for the recognition task was 13.8 (SD 3.3) in the Anodal group and 9 (SD 3.1) in the Placebo group.

Our primary endpoint measure (i.e., memory performance at Day 30) was analyzed using two-tailed independent *t*-test comparing the two groups. The experimental groups were similar on free recall performance at Day 30 [*t*(20) = 0.27, *p* = 0.78, Cohen’s *d* = 0.12, 1–β = 0.06], whereas a significant difference on hit-false alarms score between Anodal tDCS group and Placebo tDCS group was observed at Day 30 [*t*(20) = 3.36, *p* < 0.004; Cohen’s *d* = 1.49, 1–β = 0.92].

**FIGURE 3:**
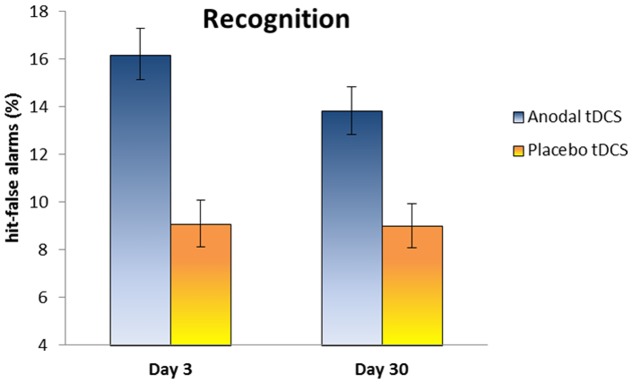
The plot shows the hit-false alarms score in the Anodal and Placebo groups at Day 3 and Day 30. Anodal tDCS improves memory recognition at Day 30 relative to Placebo tDCS. Error bars represent standard errors.

Moreover, we explored changes on memory performance at different time points (Day 3 and Day 30) with two mixed ANOVAs with “Group” (Anodal and Placebo) as the between-subjects variable and “Time” (Day 3 and Day 30) as the within-subjects variable. Regarding free recall, the analysis showed a significant effect for “Time” [*F*(1,20) = 21.6, *p* < 0.001, ηp^2^ = 0.52, 1–β = 0.99], showing a decrease of performance from Day 3 to Day 30. With respect to recognition, the analysis showed a significant effect for “Group” [*F*(1,20) = 20.4, *p* < 0.001, ηp^2^ = 0.50, 1–β = 0.99], indicating better performance in Anodal tDCS group compared to Placebo Group (see **Figure 3**).

## Discussion

The results of this study show that tDCS with the anode over the left lateral PFC strengthened existing episodic memories, an effect documented by improved recognition performance up to 30 days, relative to placebo stimulation in elderly individuals with SMC. Importantly, the observed facilitation effects was not influenced by the strategies used and the number of words correctly recalled in the last learning trial of Day 1.

The presence of behavioral effects only in the recognition tests is in line with evidence showing that the familiarity component of recognition is the relatively preserved in the aging process, whereas recollection does show age-related loss (Danckert and Craik, 2013).

Consistently with previous works (Javadi and Cheng, 2013; Sandrini et al., 2013, 2014), this study shows that the lateral PFC plays a causal role in strengthening of existing episodic memory along the lifespan. In addition, it supports previous tDCS data showing beneficial effects on memory function in older adults (Hsu et al., 2015; Tatti et al., 2016).

Long-lasting beneficial effects on episodic memory in physiological aging have been reported not only for the verbal domain (Sandrini et al., 2014, 2016) but also for visuospatial information (Floel et al., 2012; Antonenko et al., 2017). The application of anodal tDCS over the right temporo-parietal cortex, a region involved in associations between objects and locations (Sommer et al., 2005; Postma et al., 2008), during an object location memory task improved delayed free recall (Floel et al., 2012; Antonenko et al., 2017). Specifically, Floel et al. (2012) reported enhanced recall up to 1 week (offline effect) after anodal tDCS compared to placebo (sham). Conversely, no effects were observed on the learning curve and immediate free recall (online effect). These findings are consistent with previous studies showing that anodal tDCS enhanced offline, but not online effects (Reis et al., 2009; Floel et al., 2012; Santarnecchi et al., 2014; Sandrini et al., 2016), supporting the view that the consolidation processes are susceptible to anodal tDCS (Sandrini et al., 2016).

Recently, Antonenko et al. (2017) investigated the neuronal and behavioral effects of tDCS applied over the right temporo-parietal cortex during object location memory training on three consecutive days in young and older adults. Resting-state fMRI was conducted at baseline and at 1-day after training to analyze functional connectivity in the default mode network (DMN). DMN is a well-established large-scale brain network mediating episodic memory function (Jeong et al., 2015; Kim, 2016). Declines in DMN connectivity have been shown in physiological and pathological aging (Jones et al., 2011). At the behavioral level, the results showed that anodal tDCS improved memory recall, assessed 1 day after training, relative to training alone (sham stimulation). No effect on recall performance was found for the trained material at 1 month. Of note, during this follow-up assessment anodal tDCS induced beneficial effects (transfer) on a different version of the training task and a verbal episodic memory task compared to sham. Young adults performed better than older adults in all test sessions. At the neuronal level, intrinsic DMN functional connectivity increased after training in the group who received anodal tDCS. However, the lack of control sites in these tDCS studies on verbal and visuospatial episodic memories in older adults could not reveal whether only the targeted stimulation sites are critical in determining such positive effects.

Regarding the putative mechanism underlying the improvement induced by anodal tDCS in our study, facilitation of the consolidation processes could be a mechanism thought to take place in the hours or days after tDCS (Au et al., 2017). The current work and previous studies (Tecchio et al., 2010; Javadi and Cheng, 2013; Sandrini et al., 2014) showed greater consolidation after to the application of anodal tDCS during waking rest, specifically during early consolidation (Tecchio et al., 2010) or reconsolidation (Javadi and Cheng, 2013; Sandrini et al., 2014). After encoding, the reactivation of memory traces during subsequent waking state (Sirota and Buzsaki, 2005; Foster and Wilson, 2006; Karlsson and Frank, 2009; Au et al., 2017) or slow-wave sleep (Wilson and Mcnaughton, 1994; Marshall and Born, 2007) may be particular important for memory consolidation. Although highly speculative, it is conceivable that tDCS applied during waking rest, such as during early consolidation or reconsolidation, or transcranial slow-oscillations stimulation (so-tDCS) applied during slow-wave sleep (Marshall et al., 2004, 2006; Westerberg et al., 2015; Ladenbauer et al., 2016) might facilitate neural reactivation and therefore enhance systems-level consolidation for long-term retention (Au et al., 2017).

It has been shown that tDCS influences interactions between interconnected brain regions beyond the targeted area (Venkatakrishnan and Sandrini, 2012; Saiote et al., 2013). It is possible that tDCS with the anode over the left lateral PFC might have strengthened the PFC-MTL functional connectivity (Eichenbaum, 2017), therefore improving memory recognition. It might have also increased the intrinsic DMN functional connectivity (Keeser et al., 2011; Antonenko et al., 2017). The combination of tDCS with resting and task-based fMRI (Pena-Gomez et al., 2012; Shafi et al., 2012; Saiote et al., 2013) might shed light on the changes induced by tDCS after a contextual reminder in the spontaneous and task-related neuronal activity and connectivity.

The relative small sample size of this study represents a limitation and it need to be acknowledged. Further works using multiple-sessions of tDCS after a contextual reminder and larger samples should be conducted to determine the long lasting positive effects of this non-invasive intervention.

## Conclusion

This study shows for the first time that anodal tDCS over the left lateral PFC after a contextual reminder induces beneficial effects up to 30 days on verbal episodic memory in older adults with SMC. The observation that tDCS can strengthen existing memories, conceivably through reconsolidation, opens up the possibility to develop effective non-invasive interventions aimed at preventing memory decline in populations at risk of developing AD.

## Ethics Statement

This study was carried out in accordance with the recommendations of the local Human Ethics Committee of IRCCS Fatebenefratelli of Brescia, with written informed consent from all subjects. All subjects gave written informed consent in accordance with the Declaration of Helsinki. The protocol was approved by the local Human Ethics Committee of IRCCS Fatebenefratelli of Brescia.

## Author Contributions

Study concept and design: RM, MS, EG, GB, MC; Acquisition of data: RM, EG, CC, MB; Analysis and interpretation of data: RM, MS, EG, MC; Drafting of the manuscript: RM, MS, MC; Revising of the manuscript: RM, MS, EG, CC, MB, GB, MC; Statistical Analysis: RM, MS, MC; Study supervision: RM, MS, MC.

## Conflict of Interest Statement

The authors declare that the research was conducted in the absence of any commercial or financial relationships that could be construed as a potential conflict of interest.

## Footnotes

1 https://soterixmedical.com

2 www.statsoft.com
